# Therapeutic role of human hepatocyte growth factor (HGF) in treating hair loss

**DOI:** 10.7717/peerj.2624

**Published:** 2016-11-01

**Authors:** Yonghao Qi, Miao Li, Lian Xu, Zhijing Chang, Xiong Shu, Lijun Zhou

**Affiliations:** Tianjin Key Laboratory for Modern Drug Delivery & High-Efficiency, School of Pharmaceutical Science and Technology, Tianjin University, Tianjin, People’s Republic of China

**Keywords:** Hepatocyte growth factor, Transfection, Fibroblasts, Animal model, Hair follicles

## Abstract

Hepatocyte growth factor (HGF) is a paracrine hormone that plays an important role in epithelial-mesenchymal transition. HGF secreted by mesenchymal cells affects many properties of epithelial cells, such as proliferation, motility, and morphology. HGF has been reported to promote follicular growth. The purpose of the present study is to investigate the therapeutic role of HGF in hair loss treatment. A recombinant vector containing the human HGF (hHGF) gene (pTARGET-hHGF) was constructed, and the expression of hHGF in vitro was quantitatively and qualitatively evaluated. The effect of hHGF on hair growth was tested in mice, and results demonstrated that pTARGET-hHGF was successfully delivered into fibroblasts in vitro leading to a high expression of hHGF. Local injections of the pTARGET-hHGF recombinant vector into mice resulted in multiple beneficial effects compared to placebo, including faster hair regeneration, improved follicle development, and significantly increased HGF receptor (HGF-R). In conclusion, we have established a nonviral vector of hHGF which could be utilized to manipulate the sheath fibroblasts surrounding hair follicles (HF), thereby stimulating hair regeneration.

## Introduction

Alopecia is a common disease that is extremely difficult to treat in clinical dermatology. An increasing number of people are suffering from alopecia, particularly among the younger population. Some topical medicines have been utilized clinically to treat alopecia; however the therapeutic effects have been marginal or inconsistent. Therefore, development of new therapeutic approaches for treating alopecia represents a significant endeavor in clinical dermatology. Hair follicles (HF) play an important role in controlling the process of hair growth. HF are composed of epidermis and dermis. Epithelia have an inner root sheath (IRS) and an outer root sheath (ORS). Dermis consists of dermal papilla (DP) and dermal sheath (DS) (Peus & Pittelkow, 1996; Jahoda, Horne & Oliver, 1984; Jahoda & Reynolds, 1996). The growth cycle of mammalian HF, or the hair follicle growth cycle (HFGC), can be divided into three stages: anagen, catagen, and telogen (Fig. 6C). In the anagen phase, the entire HF is buried in the dermal layer of the skin to sustain the growth of the hair strand, and the DP produces the growth factors needed for hair fibers. The longer the hair stays in the anagen phase, the longer it grows. In the catagen phase, HF shrink due to disintegration and detachment of DP. During this phase, the hair is cut off from blood and other nutrition supply, and HF begins to enter the resting or the telogen phase in which they become dormant, leading to the normal process of hair loss known as shedding (Braun-Falco et al., 2000).

The hair generation process is dictated through changes in HFGC (Stenn et al., 1996; Randall, 1996). Extended HFGC could enhance the development of HF, leading to the stimulation of hair generation. Different factors, such as growth factors, cell factors, and cortical hormones during the development of HF, directly impact the progression of HFGC. Most significantly, dermal papilla cells (DPCs), the primary mesenchymal cells of DP, are quintessential in regulating HFGC. All DPCs from furs and whiskers exhibit the characteristics of aggregation growth. The most important feature of DPCs is that they can stimulate the regeneration of HF. Reynolds demonstrated that injection of DPCs cultured in vitro into the layer between dermis and epidermis of rat palm skin leads to more follicle and hair growth after the injected skin was transplanted elsewhere on the same animal (Chiu et al., 1996; Francz et al., 1993). In addition, a variety of cytokines and growth factors, as well as their receptors, are expressed in DPCs (Hibberts, Messenger & Randall, 1996; Lachgar et al., 1996; Shimaoka et al., 1995; Philpott, Sanders & Kealey, 1994; Reynolds & Jahoda, 1992; Xu & Zhou, 2014). Among those, human HGF (hHGF) has shown to be secreted by DPCs and exerts multiple biological effects on various epithelia cells (Matsumoto & Nakamura, 1996; Shimaoka et al., 1995; Xu & Zhou, 2014). hHGF is expressed in the mesenchyme and plays an important role in cell motility, proliferation, and stimulation of branching morphogenesis in the fetal lung (Ohmichi et al., 1998); however, hHGF’s role in hair regeneration remains unknown.

Our previous studies demonstrated that some Chinese herbal medicines could stimulate HF growth by promoting the proliferation of DPCs and enhancing the secretion of Hepatocyte growth factor (HGF) (Xu & Zhou, 2014). Given that hHGF could be secreted by DPCs, in the present studies, we constructed a plasmid containing hHGF gene (pTARGET-hHGF) and then transfected it into human fibroblasts. After injecting the transfected fibroblasts into mice, expression of pTARGET-hHGF and its effect in vivo were evaluated. We envisioned that the transfected hHGF could stimulate hair regeneration by directly regulating HFGC and not DPCs, thereby providing clinical evidence to support intradermal injection of plasmid and its potential application as a gene therapy for treatment of alopecia.

## Materials and Methods

### Materials

*E. coli* DH5α, *E. coli* JM109 (Promega, WI, USA), BamHI and SalI (TaKaRa, Japan), pTARGET vector (Promega, Fitchburg, WI, USA), T4 DNA ligase (Promega, Fitchburg, WI, USA), TIAN quick Midi Purification Kit, TIANgel Midi Purification Kit, EndoFree Maxi Plasmid Kit (TIANGEN BIOTECH Co., Ltd.), Polymerase Chain Reaction (PCR) primer (Shanghai Sango Biotech Co., Ltd.), human skin fibroblasts (Academy of Military Medical Sciences), EntransterTM-in vivo Transfection Reagent (Engreen), Lipofectamine™ 2000 (Invitrogen), hHGF enzyme-linked immunosorbent assay (ELISA) Kit (R&D), rabbit anti-HGF monoclonal antibody, rabbit anti-glyceraldehyde-3-phosphate dehydrogenase (GAPDH) monoclonal antibody, rabbit anti-β-catenin monoclonal antibody, Strept Avidin-biotin complex (SABC) kit and goat anti-rabbit IgG horseradish peroxidase (Wuhan Boster Biological Technology Co., Ltd.), β-catenin, HGF receptor (HGF-R) (Beijing Biosynthesis Biotechnology Co., Ltd.), Diaminobenzidine (DAB) and SP-9001 (ZSGB-BIO), Dulbecco’s modified eagle medium (DMEM) (Gibco), fetal bovine serum (FBS) (Hyclone), streptomycin sulfate and penicillin G sodium (Amresco), Boehringer DIG Luminescent Detection Kit (Boehringer–Mannheim), and Kodak X-Omat AR film (Kodak, Rochester) were commercially available and utilized according to manufacture’s protocol. The pEGFP-N1-hHGF was previously constructed in our laboratory (Fig. S1; Xu et al., 2015). Institute of Cancer Research (ICR) male and female mice (Institute of Radiation Medicine, Chinese Academy of Medical Sciences, SPF grade) were used in this study.

### Construction and identification of recombinant vector of pTARGET-hHGF

**Figure 1:**
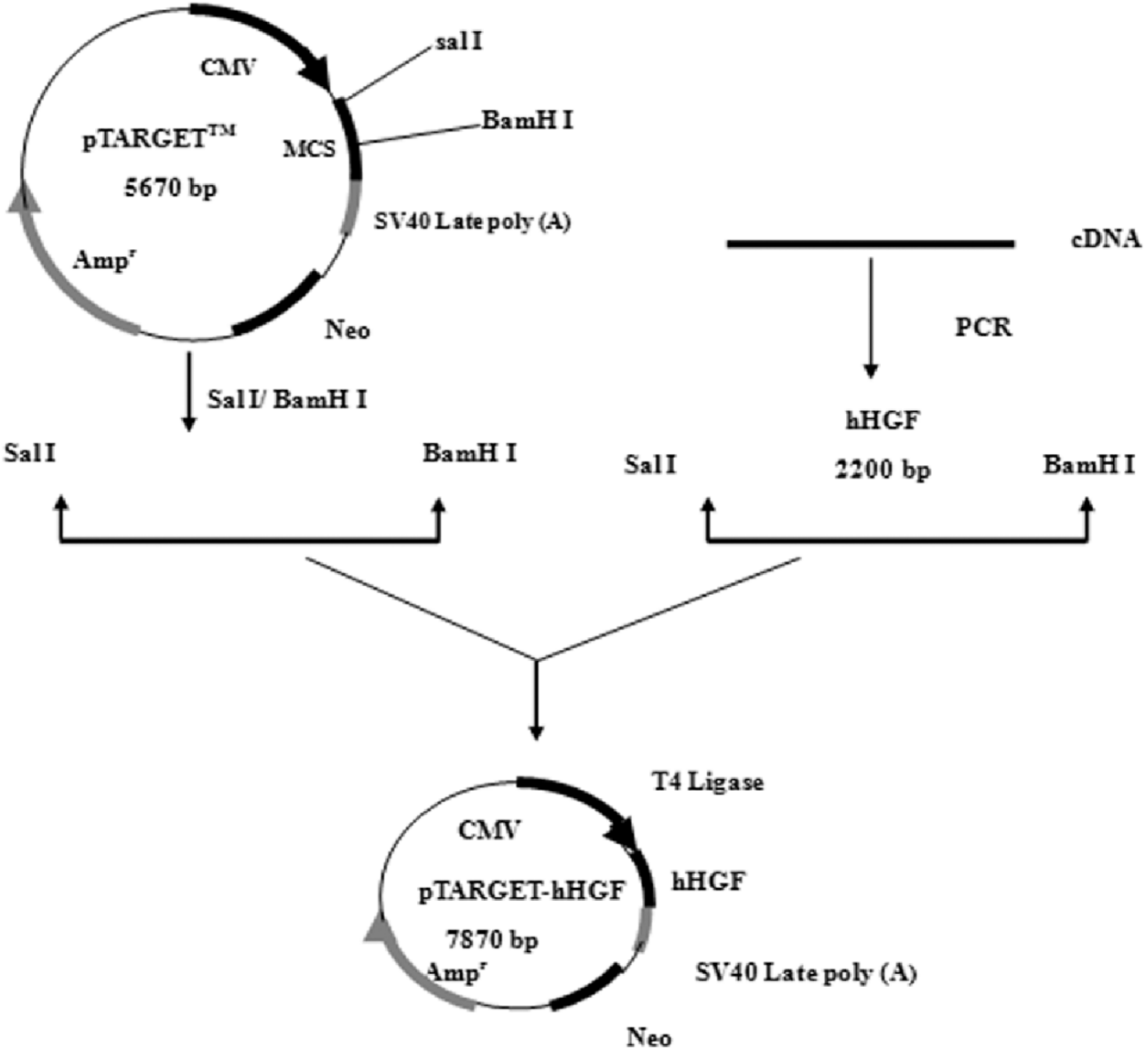
The flow chart of recombinant vector construction.

According to the hHGF cDNA sequence (NM-000601) in GenBank, the hHGF cDNA was amplified with primers (hHGF-BamHI-F: CGCGGATCCATGTGGGTGACCAAACTCC and hHGF-SalI-R: ACGCGTCGACTGTGGTACCTTATATGTTAAAATAATT). The length of the amplified segment was 2,200 bp. After purification (Endo-Free Maxi Plasmid Kit), and qualitative and quantitative analysis of hHGF with 1% agarose gel electrophoresis, hHGF cDNA and pTARGET were ligated using T4 DNA ligase as shown in Fig. 1. The ligation mixture was transformed into *E. coli* JM109, and the positive clones on LB plates containing Kanamycin-50 were then put into liquid medium and cultured at 37 °C on a shaking table. Plasmid was extracted with Endo-free Plasmid Mini Kit (OMEGA, USA). Subsequently, the extracted plasmid was digested with SalI and BamHI and separated with 1% agarose gel electrophoresis as well as colony sequencing.

### Cell culture

Human skin fibroblasts were generously provided by the Chinese Academy of Military Medical Sciences (Beijing, China). Cells were cultured in DMEM supplemented with 10% FBS. All cells were incubated at 37 °C in a humidified atmosphere with 5% CO_2_.

### hHGF plasmid transfected fibroblasts

Transfection rates of pTARGET and/or pEGFP-N1-hHGF plasmid in different concentrations of cells, DNA and/or liposome were optimized to the highest level. At a concentration of plasmid DNA: 0.8 μg/mL/well, liposome concentration of 2 μg/mL/well, and inoculated cell density in 24 well plate: 0.5 × 10^5^, 1.0 × 10^5^, 1.5 × 10^5^, 2.0 × 10^5^, 2.5 × 10^5^, 3.0 × 10^5^, 3.5 × 10^5^, and 4.0 × 10^5^ cells/mL/well. The cells were cultured in serum-free DMEM for 6 h, then replaced with DMEM supplemented with 10% FBS, and the transfection efficiency was determined 48 h later. The fluorescence emitted by the positive cells was observed with an inverted fluorescence microscope to assess transfection efficiency and expression levels of pEGFP-N1-hHGF and pTARGET-hHGF.

### Detection of hHGF protein levels in target cells

The hHGF protein expression levels in the fibroblasts were assessed with western blot analysis. Non-transfected fibroblasts and fibroblasts transfected with pTARGET-hHGF plasmid (0.8 μg/mL) were harvested, and ice-cold lysis buffer (×2) was applied to extract the protein. Samples containing equal amounts of protein were resolved by sodium dodecyl sulfate polyacrylamide gel electrophoresis (SDS-PAGE), transferred to a Polyvinylidene Fluoride (PVDF) membrane (Millipore, Billerica, MA, USA) and incubated with corresponding primary antibody (1:200 dilution) (monoclonal rabbit anti-hHGF antibody or monoclonal mouse anti-GAPDH antibody) at room temperature for 2 h, washed three times with TBST (3 × 10 min), incubated with secondary antibody (1:10,000 dilution) (anti-rabbit IgG horseradish peroxidase conjugated antibody) at room temperature for 2 h, and then washed again. The blots were developed with an enhanced chemiluminescence detection system (CWBIO). GAPDH was used as a control to assess the relative expression levels of the proteins in each group.

Cell culture supernatant was collected from cultures of fibroblasts transfected with pTARGET-hHGF plasmid at 0, 0.6, 0.8 and 1.0 μg at 48 h after transfection. ELISA was used to detect the expression of hHGF protein according to the manufacturer’s protocols. Readings from ST-360 Microplate Reader (Shanghai Kehua Bio-engineering Co., Ltd., Shanghai, China) were taken as the accurate values of experimental results, and were not corrected.

### Animal experiments

ICR male and female mice (Specific Pathogen Free: SPF) at 4 weeks of age were used in these studies. Animals were maintained in the Institute of Radiation Medicine, Chinese Academy of Medical Science. All experimental procedures using these mice were performed in accordance with relevant requirements of the guiding opinions on the treatment of experimental animals approved by the National Institutes for Food and Drug Control of China. All animal experiments have been approved by Ethics Committee on Animal Experiments of Institute of Radiation Medicine, Chinese Academy of Medical Science (The approval number is: (2016) 1-008).

Dorsal hairs were carefully removed with hair depilatory cream. Forty-eight ICR mice from which back hair was removed were randomly divided into 8 groups with each group comprised of 6 mice: pEGFP-N1-hHGF group (20, 40, and 60 μg/kg), pTARGET-hHGF group (20, 40, and 60 μg/kg), empty pTARGET plasmid control group (60 μg/kg), and negative control group. The transfection system in vivo is shown in Table 1. Mice were dosed via intradermal injection once a day for 20 d. Hair growth was recorded and photos were taken daily. Local skin samples were taken for Hematoxylin and Eosin (H/E) staining at day 8.

**Table 1:** The transfection system in vivo.

400 μL Transfection complexes	Plasmid dilution	100 μL Plasmid solution	100 μL Injection water
			50 μg DNA
		100 μL 10% Glucose solution	
	Transfection reagent diluent	100 μL Transfection reagent diluent	
		100 μL 10% Glucose solution	

### Statistical analysis

Statistical analysis was performed with SPSS software and the values expressed as the mean ± standard error of the mean. The statistical significance of differences between the control and treated groups were determined by Student’s *t-*test. Values *P < 0.05 and **P < 0.01 are considered significant.

## Results

### Identification and sequencing of the hHGF PCR amplification and pEGFP-N1-hHGF products

**Figure 2:**
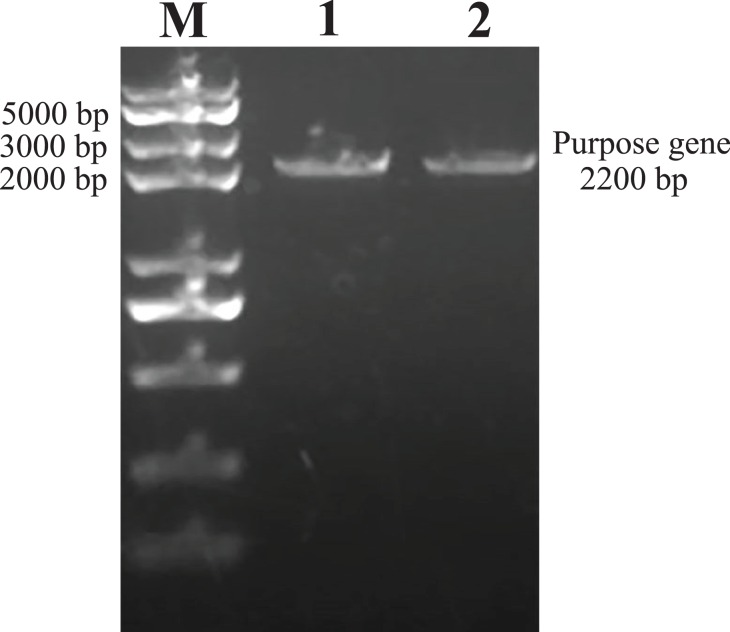
The hHGF analysis and amplification product (PCR). Lane M: Marker; Lanes 1 and 2: hHGF. The purpose gene is 2,200 bp.

**Figure 3:**
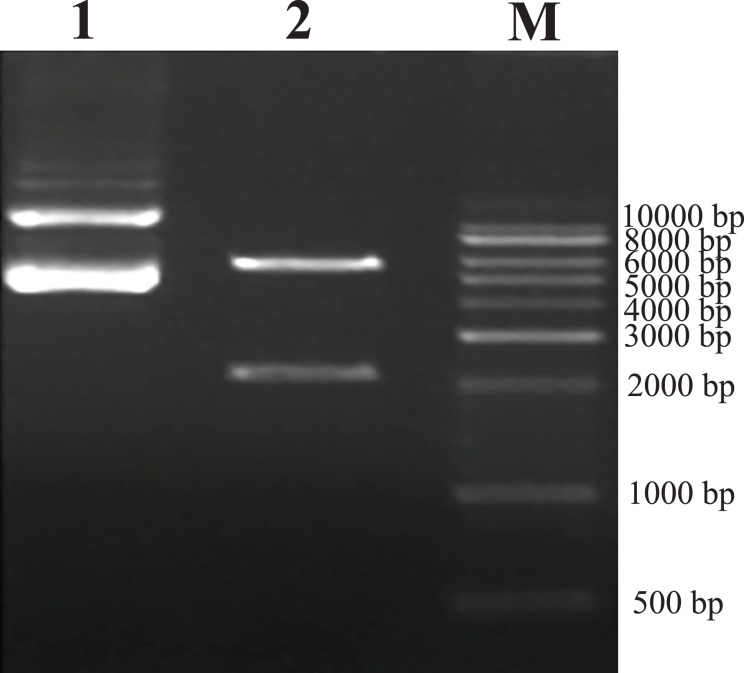
Results of double digestion. pTARGET plasmid (Lane 1) and double digested with SalI and BamHI (Lane 2).

As predicted, the hHGF product was amplified with the two primers using the pEGFP-N1-hHGF plasmid at approximately 2,200 bp on a gel (Fig. 2). After the pTARGET-hHGF clone was double digested with SalI and BamHI, both hHGF band (approximately 2.2 kb) and vector band (approximately 5.67 kb) were observed (Fig. 3). The clone was submitted for sequence analysis. The base sequence of the inserted gene in the pTARGET plasmid matched the genomic sequence of hHGF in GenBank. The final plasmid was termed pTARGET-hHGF.

### Production and titration of the recombinant lentiviral vector

**Figure 4:**
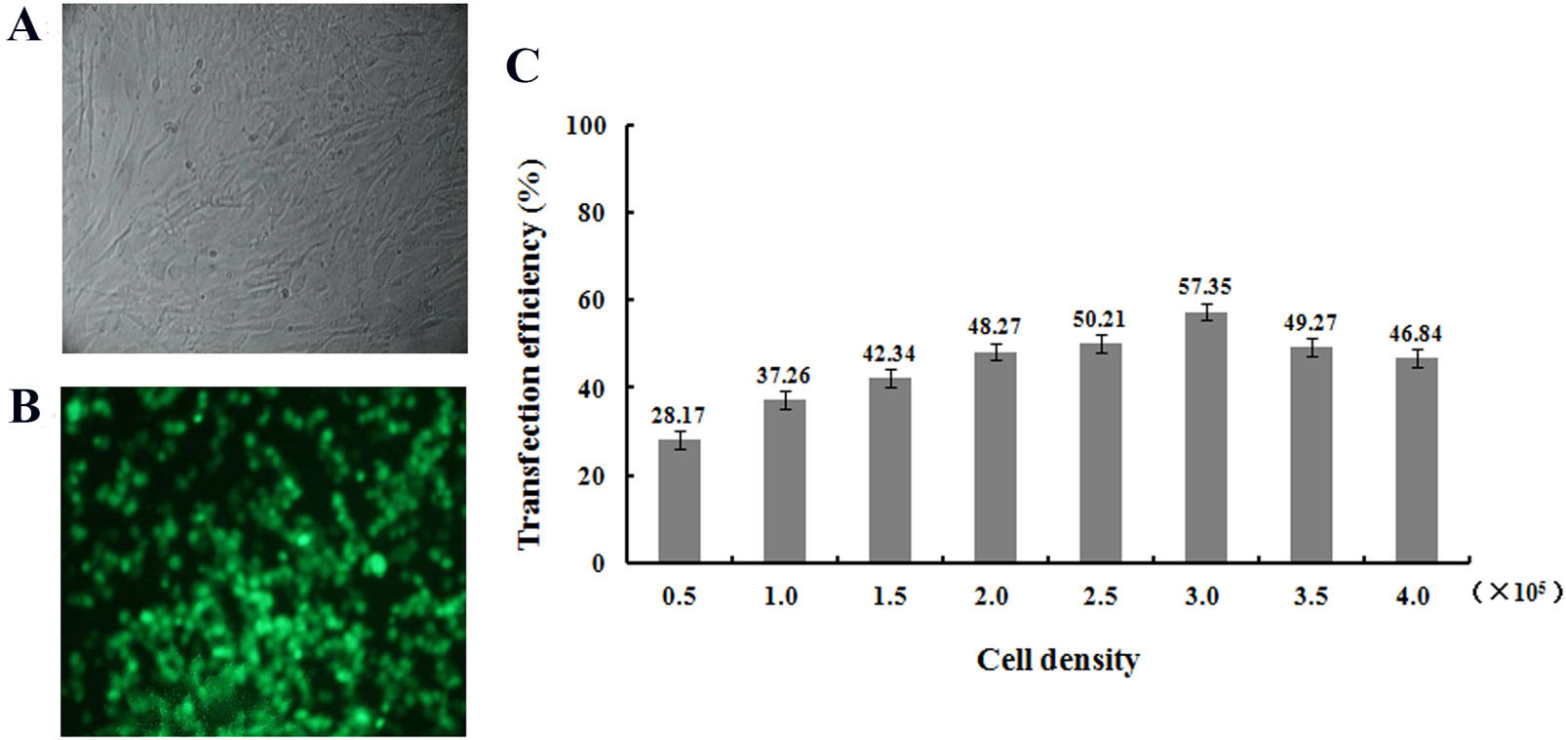
Transfection results. Green fluorescent protein (GFP) was observed to determine the expression of pEGFP-N1-hHGF plasmid in fibroblasts after transfection for 48 h (100×) ((A) bright field; (B) fluorescence). (C) Cell seeding density was optimized through examining the transfection ratio at a concentration of plasmid DNA: 0.8 g/well, liposome concentration: 2 μg/well (24 well plate).

After transfection of fibroblasts with the pEGFP-N1-hHGF and pTARGET-hHGF plasmids, Green Fluorescent Protein (GFP) expression from pEGFP-N1-hHGF was assessed using a fluorescence microscope to ensure that the plasmids were properly generated (Fig. 4A). As analysis indicates that when the concentration of plasmid DNA is 0.8 μg/well and liposome concentration is 2 μg/well, the optimal cell seeding density is 3.0 × 10^5^ cells/mL/well (Fig. 4B).

### hHGF protein expression in fibroblasts

**Figure 5:**
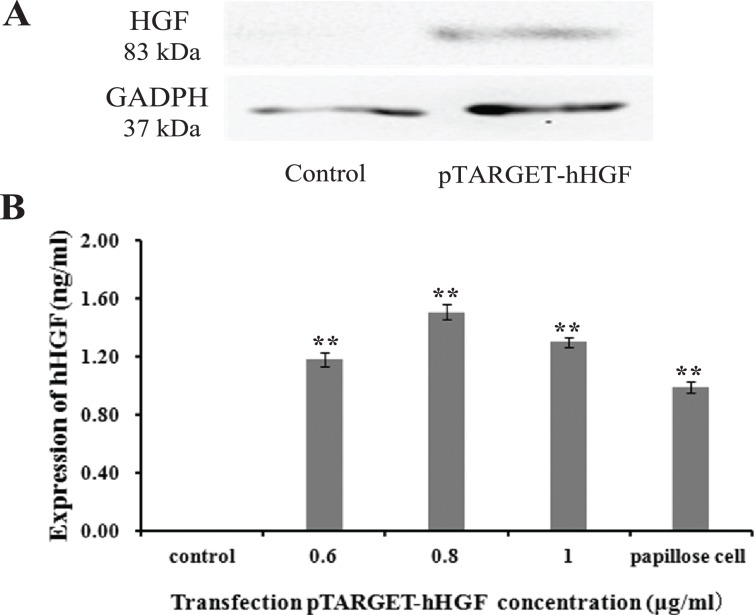
The hHGF protein expression in fibroblasts after transfection with the pTARGE T-hHGF plasmid. (A) Expression of hHGF as assessed by Western blot. (B) ELISA of hHGF in the fibroblasts supernatants after the transfection at different DNA concentrations.

Protein lanes corresponding to the non-transfected fibroblasts and the fibroblasts transfected with pTARGET-hHGF plasmids are shown in Fig. 5A. The GAPDH protein ran at 37 kDa and the hHGF protein at 83 kDa. The non-transfected fibroblasts did not express hHGF, while the hHGF-transfected group expressed hHGF. ELISA showed that pTARGET-hHGF infected fibroblasts produced hHGF protein, and that the concentration was higher in the hHGF infected group at DNA concentration at 0.8 μg/well (P < 0.01) (Fig. 5B).

### Animal experiments

**Figure 6:**
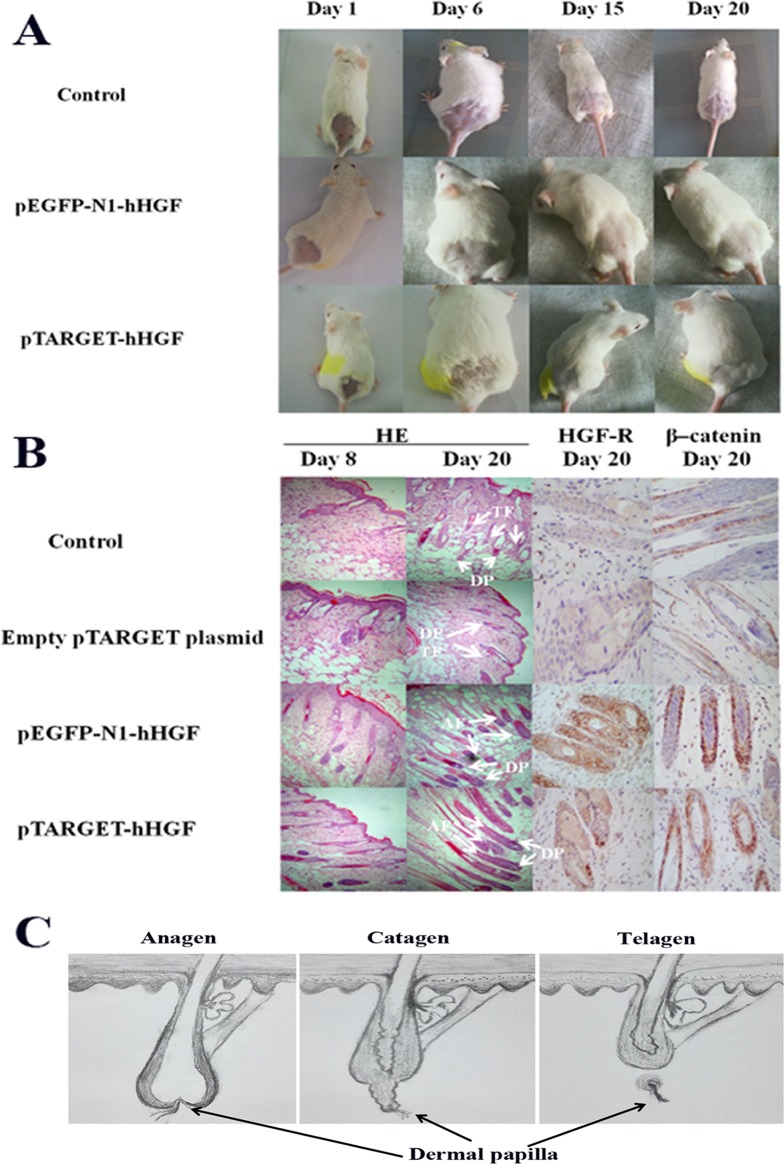
Animal experiments. (A) Hair growth of the pTARGET-hHGF group, the pEGFP-N1-hHGF group, and the negative control group on day 1, day 6, day 15 and day 20. The pTARGET-hHGF and pEGFP-N1-hHGF-treated group showed the beginning of hair growth from day 6, while the control group did not exhibit any growth. (B) Local skin tissue sections of mice, the staining of HE, HGF-R, and β–catenin. For mice tissue sections at day 8 and day 20, HE staining showed tissue sections of dermal of pTARGET-hHGF and pEGFP-N1-hHGF groups began the growth of HF at day 8, while the negative or empty plasmid control group showed almost no HF growth. In addition, the expression of HF in the anagen phase of pTARGET-hHGF group and pEGFP-N1-hHGF groups was much higher than the control group at day 20 (TF, Telogen follicle; AF, Anagen follicle; and DP, Dermal papilla). For HGF-R and β–catenin immunohistochemical staining, both pTARGET-hHGF and pEGFP-N1-hHGF groups had significant positive staining in HF (Brown staining is positive results), while HGF-R staining was almost negative in the control group, and there were only a few positives in the β–catenin staining for the control group. The doses of vector were 60 μ�g/kg. (C) Hair follicle growth cycle.

ICR mice were used to investigate the effects of pTARGET-hHGF and pEGFP-N1-hHGF on the hair growth. The dorsal hair of ICR mice was removed and then the resulting mice were injected with various concentrations of pTARGET-hHGF or pEGFP-N1-hHGF. Results demonstrated that the hair growth rate in the dorsal skin of the ICR mice treated with pTARGET-hHGF or pEGFP-N1-hHGF (Injection concentration was 60 μg/kg) was higher than that of the negative or empty plasmid control group (Fig. 6A). H/E staining showed that at the beginning of day 8, pTARGET-hHGF and pEGFP-N1-hHGF groups experienced a significant increase in total number of HF when compared to the control or empty plasmid group (Fig. 6B; Table 2). Furthermore, immunohistochemical staining showed a significant increase of HGF-R and β-catenin in HF for the pTARGET-hHGF and pEGFP-N1-hHGF groups, when compared with the control or empty plasmid group (Fig. 6B).

**Table 2:** The quantity of HF.

	Group			
Day	Control	Empty plasmid	pEGFP-N1-hHGF	pTARGET-hHGF
Day 8	1.21 ± 0.09	1.52 ± 0.10	10.96 ± 1.45**	11.02 ± 1.02**
Day 20	10.01 ± 1.33	10.26 ± 1.55	15.67 ± 0.98*	19.86 ± 1.27*

**Notes:**

n = 6.

* P < 0.05.

** P < 0.01.

## Discussion

Many physiological events are involved in the growth and development of HF, such as interaction between dermis and epidermis, as well as development and differentiation of the cells. HF play an important role in skin self-repair, wound healing, and onset of carcinoma. The dermal parts of HF consist of DPCs and DS fibroblasts which have the ability to induce the formation of HF through the interaction between epithelial cells and the mesenchyme cells of HF. DPCs are a composite of very unique fibroblast cells that produce an extracellular matrix (ECM) and show great significance in the interaction between epithelial cells and stromal cells (Matsuzaki & Yoshizato, 1998; Almond-Roesler et al., 1997). The cause for hair loss is the imbalance between the anagen and telogen phases of HFGC. In the telogen phase, the ECM of DPCs is reduced, and epithelial cells of the hair bulb are narrowed and separated from the DPCs. In the catagen phase, DP remains close to the base of HF to form cells that are closely bunched and embedded in the ECM, leading to an unrecognizable ultra-structure (Stenn & Paus, 2001; Pans & Cotsarelis, 1999). Thus, the DP plays an important regulatory role in HFGC, and hair loss is associated with DP dysfunctions.

Our previous work illustrated that certain Chinese herbal medicines can stimulate HF growth by promoting the proliferation of DPCs and enhancing the secretion of hHGF (Xu & Zhou, 2014; Xu et al., 2015), thereby stimulating HF growth. In addition to our study showing hHGF’s role in promoting growth of HF, other recent studies have revealed that hHGF can also (a) regulate interactions between DP cells and epithelial keratinocyte and other interactions among cells (Fujie et al., 2001; Lindner et al., 1997); (b) induce the formation of per follicular blood vessels (Houseknecht et al., 1998; Boulton, 1999; Zhang et al., 2003; Mecklenburg et al., 2000); and (c) influence all three phases on the HFGC of hairs (Jindo et al., 1998). Importantly, our study revealed that while DPCs can secrete hHGF, the surrounding sheath fibroblasts of HF cannot (Xu et al., 2015).

Consequently, we hypothesized that manipulating sheath fibroblasts to mimic functional DPCs to express hHGF could promote the growth of HF. Based on this understanding, we (a) first constructed a recombinant plasmid expression vector containing hHGF (pTARGET-hHGF); (b) demonstrated that this plasmid could be transfected into the surrounding sheath fibroblasts and that it could secrete hHGF in vitro; and (c) pursued animal studies using mice to show that it could promote hair growth as well as tissue follicle development.

The hHGF expression indeed increased by more than 57% when fibroblasts were transfected the recombinant plasmid. The hHGF gene transfected fibroblasts expressed hHGF at even higher levels than DPCs. These results demonstrate that the recombinant plasmid vector successfully delivered the hHGF gene into sheath fibroblasts which then expressed the hHGF gene at a high level. This transformation yields sheath fibroblasts with the function of DPCs. Further animal experiments showed that both pTARGET-hHGF and pEGFP-N1-hHGF groups regenerated mouse hair faster than the control group. Moreover, H/E staining of local skin tissue sections from mice showed that growth of HF started at day 8 for both the pTARGET-hHGF group and pEGFP-N1-hHGF group, while the control group had almost no signs of HF growth.

The examination of the expression of HGF-R in mouse skin tissue sections is noteworthy because hHGF achieves its functions through binding to its specific receptor HGF-R. HGF-R immunohistochemical staining showed that pTARGET-hHGF and pEGFP-N1-hHGF groups had significant positive staining in HF, while such staining was almost absent in the control group. This outcome suggests that the active hHGF had stimulated the growth of HF in both pTARGET-hHGF and pEGFP-N1-hHGF groups, thereby further validating the observation that hHGF recombinant vector was transfected into the cells surrounding the HF of mice, leading to secretion of active hHGF.

It has been reported that β-catenin, a ubiquitously distributed protein with multiple functions, plays an important role in controlling the HF morphogenesis and stem cell differentiation in the skin (Huelsken et al., 2001). If β-catenin is deleted after HF have formed, hair is completely lost after the first hair cycle (Wang et al., 2000). β-Catenin is also essential to the fate of skin stem cells because, in the absence of β-catenin, stem cells fail to differentiate into follicular keratinocytes instead adopting an epidermal fate (Huelsken et al., 2001). Our experimental results indicate that hHGF enables β-catenin to enter the nucleus in cultured epithelial hair follicle stem cells (Fig. S2). At the same time, β-catenin immunohistochemical staining for both pTARGET-hHGF and pEGFP-N1-hHGF groups showed a positive staining that is significantly stronger than the negative control group in HF (Fig. 6B). These results suggest that hHGF may promote growth of HF by increasing the expression of β-catenin. In addition, liver and kidney tissue sections did not show fluorescence in the pEGFP-N1-hHGF local injection group, thereby demonstrating the potential clinical safety of the hHGF recombinant plasmid expression vector.

Finally, Lindner et al. (2000), suggested that hHGF accelerates HF morphogenesis and inhibits catagen induction (thus extending the anagen phase) by analyzing hHGF transgenic 8-week old C57BL/6 mice and treating HF with recombinant hHGF protein. Müller-Röver et al. (2001) achieved a similar experimental outcome in first neonatal C57BL/6 mice. Our experiments employed 4-week old ICR mice with dorsal hairs removed using hair depilatory cream to reach synchronized HFGC, but still showed that hHGF promotes the growth of HF. These results strongly suggest that hHGF can accelerate HF morphogenesis and growth in different mouse strains and at different ages.

## Conclusion

In summary, we report here construction of a recombinant vector, pTARGET-hHGF, that contains the hHGF gene, and that this new recombinant vector could be successful delivered into fibroblasts in vitro. We have found that such transfection leads to secretion of hHGF, and a high level of the hHGF expression could be detected qualitatively and quantitatively, thereby allowing fibroblasts to possess functions of DPCs. Furthermore, local injections of pTARGET-hHGF into live mice can result in faster hair regeneration, improved follicle development, and significantly increased expressions of HGF-R and β-catenin. These results demonstrate that we have established a nonviral vector of hHGF that can manipulate the sheath fibroblasts surrounding HF via hHGF expression, thereby promoting hair regeneration.

## Supplemental Information

10.7717/peerj.2624/supp-1 Supplemental Information 1 Result of the double digestion. (A) Double digestion result of pEGFP-N1. Lane M: Marker; Lanes 1 and 2: pEGFP-N1. (B) Double digestion result of recombinant plasmid pEGFP-N1-hHGF. Lane M: Marker; Lane 1: Recombinant plasmid pEGFP-N1-hHGF.

10.7717/peerj.2624/supp-2 Supplemental Information 2 The expression level of β–catenin in hair follicle stem cells. (A) Before any stimulation with hHGF, hair follicle stem cells expressed β–catenin mainly in the cell membrane and cytoplasm. (B) After stimulation with hHGF for 15 min, hair follicle stem cells expressed more β–catenin in the nucleus than in the cell membrane and cytoplasm. (C) After stimulation with hHGF for 30 min, much more β–catenin was expressed in the nucleus. (D) K15 staining. (E) K19 staining.

10.7717/peerj.2624/supp-3 Supplemental Information 3 PCR reaction for HGF cDNA amplification.

10.7717/peerj.2624/supp-4 Supplemental Information 4 The ligation reaction for the recombinant.

## Additional Information and Declarations

### DNA Deposition

The following information was supplied regarding the deposition of DNA sequences: GenBank accession number: NM-000601.

### Data Deposition

The following information was supplied regarding data availability: The raw data has been supplied as Supplemental Dataset Files.
