# Supplementary material for: Therapeutic role of human hepatocyte growth factor (HGF) in treating hair loss
Source: PeerJ. 2016 Nov 1;4:e2624. doi: 10.7717/peerj.2624 (PMC5101615; doi:10.7717/peerj.2624)
Supplement: Supplemental Information 3 [file peerj-04-2624-s003.docx]

| Components | Volume |
| --- | --- |
| Template DNA  Former primer (10 μM)  Reverse Primer (10 μM)  5×TransStart FastPfu Buffer  2.5 mM dNTPs  TransStart FastPfu DNA Polymerase  ddH_2_O to final volume | <0.5 μg  2 μL  1.5 μL  10 μl  1.25 μl  1 μl  50 μl |

Table S1. PCR reaction for HGF cDNA amplification
