# Supplementary material for: Therapeutic role of human hepatocyte growth factor (HGF) in treating hair loss
Source: PeerJ. 2016 Nov 1;4:e2624. doi: 10.7717/peerj.2624 (PMC5101615; doi:10.7717/peerj.2624)
Supplement: Supplemental Information 4 [file peerj-04-2624-s004.docx]

| Components | Volume |
| --- | --- |
| HGF cDNA (2 μg/ml)  10×T4 ligase buffer  T4 DNAligase  pTARGET vector (60 μg/ml) | 4 μl  2 μl  1 μl  3 μl |

Table S2. The ligation reaction for the recombinant
